# Clinical diagnosis, prevention, and treatment of neurodyspepsia syndrome using intelligent medicine

**DOI:** 10.1515/biol-2022-0802

**Published:** 2024-05-07

**Authors:** Jingyu Zhu, Wei Meng, Liang Liu, Peixin Hu, Yuling Liang, Wenwen Zhu, Xiaoyan Zhu

**Affiliations:** Department of Gastrointestinal Endoscopy, Central Hospital Affiliated to Shandong First Medical University, Shandong, China

**Keywords:** intelligent medical treatment, neurodyspepsia syndrome, internet of things technology, clinical diagnosis

## Abstract

Against the backdrop of rapid social economy and scientific and technological development, intelligent medical technology expanded based on the Internet plays a crucial role in the innovation and development of the modern medical industry. Intelligent medical technology has completely changed the fixed medical methods of the past, and it can solve the isolated defects between various unit systems, greatly improving the overall informatization level of hospitals. This article analyzed the clinical diagnosis, prevention, and treatment of neurodyspepsia syndrome (NDS) in intelligent medicine. Dyspepsia can cause palpitations, vomiting, abdominal distension, dizziness, and other symptoms so that it can cause discomfort and pain in the middle or around the epigastric region. Therefore, it is necessary to make a correct diagnosis of neurodyspepsia in order to reduce the discomfort of patients. Intelligent medical technology is of great significance in improving patients’ symptoms. This study sets up a control group and an experimental group for the experiment. The control group used conventional medication technology, while the experimental group used intelligent medical technology to analyze the patient samples taken. By comparing the factors that affect patients with NDS, it was found that the physical function score of the experimental group was 6.3% lower than that of the control group. Intelligent medical technology has high diagnostic efficiency and can achieve rapid diagnosis of NDS, meeting the clinical diagnosis and prevention requirements of NDS.

## Graphical abstract

**Figure:**
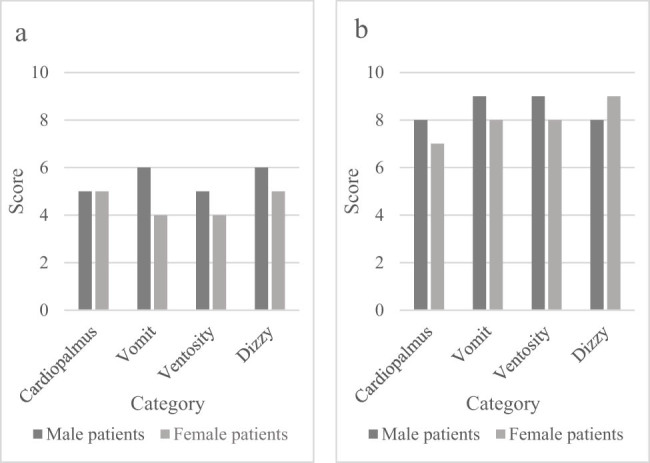

## 1 Introduction

Neurodyspepsia syndrome (NDS) is a common digestive disease, which is a series of symptoms caused by dysfunction of the upper gastrointestinal tract. The clinical manifestations of patients include palpitations, vomiting, abdominal distension, dizziness, etc. These symptoms usually last for more than 1 month. Previously, patients would use symptomatic regimens for treatment, but if only a single drug were used, its efficacy would be difficult to satisfy. In recent years, people’s understanding of this disease has become increasingly profound, and some studies have shown that the therapeutic effect of intelligent medicine has gradually become prominent, which can quickly detect digestive tract diseases.

Researchers in relevant fields have conducted research on indigestion syndrome and analyzed the causes of the patient’s illness. Burns analyzed the obvious symptoms that dyspepsia syndrome may exhibit, but due to the unclear molecular mechanism of gastrointestinal tissue and the need for further research on related inflammatory reactions, he drew on the symptoms and treatment plans of diseases related to “functional gastrointestinal disease,” “functional dyspepsia,” and “irritable bowel syndrome.” By analyzing the changes in the number of lymphocytes in the patient’s digestive tract, it was found that the imbalance of the digestive tract environment is an important reason for clinical manifestations [1]. Hanel et al. analyzed the incidence and mortality rate of indigestion syndrome and found that the incidence and treatment effect of indigestion syndrome were also on the rise. They used bioinformatics technology to study indigestion syndrome. By analyzing patients’ psychological factors, hormone levels, and changes in physical function, they found that some factors can lead to the occurrence of functional gastrointestinal diseases [2]. Koloski found potential immune activation and psychological distress in patients with indigestion syndrome. In a large-scale population-based study, he tested whether allergic or autoimmune diseases were associated with indigestion syndrome. The results indicated that testing for allergic or autoimmune diseases is associated with NDS [3]. Shah evaluated and compared the impact of antimicrobial therapy on patients with digestive syndrome. He measured the symptom response to standardized nutrition challenges based on the total score of gastrointestinal symptoms measured through questionnaires and subscales. After experimental analysis, it was found that the subscale of indigestion and diarrhea showed significant improvement in statistics. Similarly, symptom scores significantly improved after standardized nutrition challenges [4]. These authors have provided some suggestions and conclusions by analyzing indigestion syndrome.

In addition, the authors analyzed the factors that influence indigestion syndrome and obtained data on each influencing factor through investigation. Talley analyzed whether dyspepsia syndrome is associated with smoking and evaluated whether smoking is an independent risk factor for the disease. He evaluated patients during clinical visits using an abdominal symptom questionnaire. In the data meta-analysis, intelligent medical technology was used to analyze the impact of smoking on symptom status, and adjustments were made for smoking, age, and gender. It was found that smoking is an important environmental risk factor for indigestion syndrome [5]. Tack et al. analyzed the key pathological factors of indigestion syndrome and found that the use of first-cause drugs is often considered the preferred treatment method for the disease. Experimental analysis showed that first-cause drugs may be effective and safe for patients, but there is a lack of available and proven effective drugs [6]. Barberio analyzed the severity of symptoms of indigestion syndrome and data on mental health and found that in patient’s rehabilitation reports, the use of multiple medication methods is more effective. The treatment effect of using only one drug is poor, laying a foundation for future prevention, control, and diagnosis of digestive tract diseases [7]. However, the relevant content investigated by these researchers is insufficient to effectively address indigestion syndrome, and improvements in treatment plans and diagnostic techniques are needed.

This article analyzed the clinical diagnosis, prevention, and treatment methods of NDS using intelligent medical technology, and analyzed intelligent medical technology. The application of intelligent medical technology in the medical field was discussed, and the clinical diagnosis, prevention, and treatment plans for NDS were studied. This article applied intelligent medical technology to the diagnosis of NDS. Through experimental research, it was found that intelligent medical technology can more quickly treat and prevent NDS.

## 2 Intelligent healthcare

### 2.1 Current status of intelligent medical development

In the future, more high-tech technologies such as artificial intelligence and sensing technology will be introduced into the medical field, making medical services more intelligent and promoting the vigorous development of medicine. With the implementation of the new “medical reform” policy, intelligent healthcare has gradually entered people’s daily lives. As the birth rate of the population continues to decline, people are paying more and more attention to their physical condition, and people have higher and higher requirements for the health system. So, the combination of telemedicine and electronic medicine can bring great convenience to life. By utilizing technologies such as cloud computing and artificial intelligence expert terminals, a complete Internet of Things (IoT) health management system can be established, enabling everyone to enjoy first-class health management.

When IoT technology is applied to the medical field, the Drug Administration will take many practical measures to promote the implementation of these policies. Intelligent healthcare would be fully utilized in medical services, including medical information interconnection, medical service sharing, medical service innovation, and public health prevention and control. By providing a deeper level of service efficiency and service quality in intelligent healthcare, the overall management of the hospital is improved, and monitoring is brought to a wireless level, thus completely transforming and solving modern digital healthcare issues and problems. It greatly improves the sharing of medical resources and reduces public medical expenses.

By utilizing electronic medicine and IoT technology, a large amount of medical monitoring work can be wirelessly realized. Remote medicine and self-service medicine can collect and highly share information in a timely manner, thereby alleviating the difficulties of resource shortage and uneven distribution and reducing public medical expenses.

The development of intelligent medical services can be divided into seven levels: the first level is operational management, which mainly includes information such as hospital expenses and drugs; the second is an electronic medical record system composed of patient data and imaging data; the third is its clinical application, mainly including computer input of doctors’ medical orders, etc.; the fourth is to establish a governance system for chronic diseases; the fifth is to establish a regional medical and health information exchange system; the sixth is a medical assistance decision-making system; and the seventh is to establish a universal healthcare system. In the field of remote intelligent healthcare, some more developed hospitals have made technological improvements in mobile information applications, which would play a driving role in future intelligent healthcare.

### 2.2 Application of IoT technology in intelligent healthcare

Through the introduction of IoT technology, hospitals can track the location of patients, drugs, and medical waste. By utilizing portable mobile phones with wireless network access, patients’ conditions can be more accurately, timely, and comprehensively understood. By utilizing this system, real-time monitoring of the ward can be achieved. It can monitor the elderly and patients in real time and help them grasp their vital signs in a timely manner. “Remote medicine” is a new type of medical service. The organic integration of medical technology with computer technology, multimedia technology, and Internet technology can improve the effectiveness and quality of treatment and can reduce the cost of medicine, thus achieving a better response to people’s needs for health and medicine. On the basis of IoT technology, the construction of hospital informatization has become particularly crucial. Utilizing modern IoT technology and relevant terminal devices enables real-time tracking and monitoring of patient signs. This enables real-time diagnosis and health alerts for patients or subhealthy patients in hospitals, effectively reducing and controlling their diseases and thus improving work efficiency. Throughout the entire treatment process, reducing material consumption can reduce medical accidents and improve medical quality.

## 3 Neurogenic dyspepsia syndrome

### 3.1 Main symptoms of indigestion

As a common digestive disorder, dyspepsia is associated with the development of receptive relaxation of the gastric fundus, visceral hypersensitivity, and gastrointestinal motility disorders after diet, as well as environmental, social, and spiritual factors. In recent years, due to the rapid development of life and the increasing speed of life, there have been significant changes in lifestyle and dietary structure. In addition, the stress of work has led to a gradual increase in the incidence of this disease.

The symptoms of indigestion are closely related to gastric syndrome and gastric spasms. Gastrointestinal symptoms typically manifest as delayed digestion in the stomach. During treatment, patients with indigestion are reclassified and differentiated based on the severity of the symptoms [8]. Dyspepsia syndrome is related to the quality of life and psychological status of patients. The symptoms of outpatient patients in tertiary hospitals mainly include diarrhea and constipation. These gastrointestinal symptoms affect the evaluation of the causes of patients’ illnesses, and psychological factors are important factors affecting gastrointestinal diseases [9].

The symptoms of indigestion vary in the digestive system and gastrointestinal tract, and doctors provide targeted treatment for potential patients. Functional dyspepsia can provide clinical experience for the study of overlapping gastrointestinal diseases. It has many clinical reactions, such as residual returning flow in the esophagus, postprandial pain syndrome, and digestive discharge difficulties. The study of the above symptoms provides a basis for the physiology of pathology [10]. Esophageal duodenoscopy in children found that more than half of cases with NDS showed signs of stomach disease, and destructive ulcerated lesions were diagnosed in almost all age groups. The frequency of such lesions increased in adolescents [11].

From the perspective of traditional Chinese medicine, gastrointestinal dysfunction can be classified into the categories of pathogenesis, such as “epigastric pain” and “gastric distension.” In traditional Chinese medicine theory, changes in mood, improper diet, and weather conditions lead to a complex pathological process of liver fire and spleen stomach disharmony. Dyspepsia is a common disease in clinical practice, characterized by a long course of disease and a tendency to recur. If the patient’s quality of life is poor, effective measures must be taken to help them recover.

Liver depression and spleen deficiency-type dyspepsia syndrome are also a common disease, and their etiology is not yet fully understood. Through clinical pharmacological analysis of traditional Chinese medicine, it was found that Shugan Jianpi Tang is suitable for the clinical symptoms of gastrointestinal diseases with liver stagnation and spleen deficiency, thereby helping patients recover as soon as possible.

In addition, modern pharmacological studies have shown that “bergamot heart” has anti-depression, antibacterial properties, and certain protective effects on gastrointestinal cells. Fructus Aurantii and Radix Bupleuri have the effect of improving intestinal emptying and intestinal function, especially since Radix Bupleuri has anti-inflammatory and gastrointestinal regulating effects. Some of the therapeutic effects of other drugs are very good. This article analyzed the causes of this phenomenon and believed that the main reasons are the following: these drugs can regulate the central nervous system of the hypothalamus and enhance the vagus nerve; at the same time, they can also promote the secretion of gastric juice. The causes of indigestion include increased gastric acid secretion caused by sympathetic nerve stimulation and imbalance of vagal sympathetic inhibition, resulting in stomach disease with liver stagnation and spleen deficiency. Through the cerebral cortex, the cerebral cortex center and hypothalamus are abnormal, so the sympathetic nerve in the brain is hyperactive. After that, its release pulse is enhanced, which lasts for a long time, and finally, the digestive tract is abnormal. Based on the analysis of traditional Chinese medicine, clinical symptoms are alleviated and contribute to rehabilitation.

### 3.2 Diagnosis and treatment plan for indigestion syndrome

#### 3.2.1 Diagnosis of neurodyspepsia

Neurodyspepsia can cause damage to any segment of the digestive tract along the gastric wall, starting from the brainstem. Along the farthest segment, it can cause damage to the vagus nerve and its branches, leading to inflammatory reactions or dysfunction. The above situations can cause gastrointestinal dysfunction and are classified as indigestion. According to the location and severity of the damage, its clinical symptoms vary. Generally, neurodyspepsia manifests as intermittent or persistent, often associated with functional overflow obstruction or vomiting symptoms.

Abdominal ultrasound is of great help in the diagnosis of neurodyspepsia. Ultrasound can help determine the characteristics of abdominal fluid and whether there is fibrosis or abscess, as well as determine the size of organs and other similar tumor diseases. However, currently, the sensitivity and specificity of clinical diagnosis for neurodyspepsia are low; therefore, abdominal ultrasound is the best auxiliary examination method. When the equipment is complete, X-ray images of the gastric reticulum are very useful for detecting perforated foreign bodies in the gastric reticulum. X-ray images of the larynx and chest are also useful for the diagnosis of diseases in the larynx and chest, and cytological testing of tumor cells is performed after peritoneal puncture.

Neurodyspepsia includes symptoms such as dyspepsia and irritable bowel syndrome. By investigating the impact of symptom overlap on the burden of symptoms in NDS, it is found that overlapping dyspepsia in NDS is related to different clinical and psychological characteristics. By screening patient data from the population, it is possible to compare the treatment effects of subjects who use drugs alone and those who overlap two or three drugs [12]. By analyzing the gastric regulation of patients after meals, doctors can find that gastric fundus regulation may lead to a temporary increase in relaxation of the lower esophageal sphincter, leading to gastroesophageal reflux. Functional dyspepsia, as a common disease, affects the normal life of patients. In diagnosis, endoscopic examination of the digestive tract is required, and inhibitors can be used to treat the disease [13].

**Figure 1:**
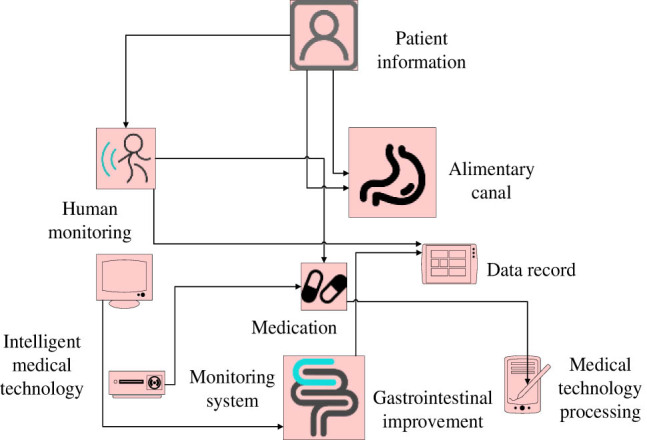
The treatment plan of intelligent medicine for NDS.

Medication is administered in a population of children with postprandial pain syndrome due to neurodyspepsia, and clinical reactions and safety are observed. Clinical reactions are divided into complete response, partial response, and no response. The prescribed dosage, side effects, and weight changes during medication treatment can all affect the observed results. The most common adverse reactions are weight gain and restlessness. Medication therapy is an option for treating functional nausea and dyspepsia syndrome in children and adolescents, especially for patients with concurrent weight loss, anxiety, and insomnia [14]. The neural mechanisms of gastrointestinal interactions and visceral hypersensitivity reactions are closely related. The use of advanced imaging techniques such as magnetic resonance imaging for diagnosis has been recognized by clinical doctors and radiologists in the diagnosis of gastrointestinal diseases. The research results on the population of patients with NDS demonstrate that psychological and social factors affect the mechanism of regulating visceral sensitivity in the brain. Psychological processes can affect the function of the digestive system and may lead to symptoms of indigestion. The mental state related to stress in patients can affect the processes that occur in the central nervous system and trigger somatic reactions in the digestive tract through the autonomic visceral system [15]. The treatment plan for NDS using intelligent medicine is shown in Figure 1.

#### 3.2.2 Treatment plan for neurodyspepsia

Some primary symptoms of neurodyspepsia, such as tumors, gastric torsion, and stomach pain caused by peritonitis, are often difficult to treat [16,17]. Primary diseases such as pharyngeal trauma, severe pneumonia, and other injuries that cause vomiting disorders only require treatment, such as pharyngitis and cellulitis, which can be treated with oral broad-spectrum antibiotics, anti-inflammatory drugs, and analgesics [18]. For the above patients, if they have refractory vomiting symptoms, a rumen fistula can be established to alleviate long-term vomiting symptoms, and then diet and standing water treatment can be carried out through this method [19]. In valuable diseases, if the cause is in the abdomen, surgical treatment is necessary. For some primary causes of neurodyspepsia, the best approach is to undergo open surgery and gastrotomy. This not only helps doctors diagnose and judge the condition clearly, but also helps patients empty food from the stomach, thereby reducing the pressure on nerve receptors [20].

When this damage is reversible, it can restore the stomach to a normal contractile state. Before surgery, intravenous infusion is needed to restore water in the body and adjust the pH and electrolytes in the body. If it may be caused by peritonitis, timely antibiotic treatment is necessary. Due to the functional emptying of the gastrointestinal tract, oral liquid supplementation or oral medication should be prohibited [21,22]. When there is low calcium in the blood, patients should be injected with calcium to compensate for nutrient loss caused by decreased absorption in the digestive tract. When performing surgery, it is important to conduct a complete and thorough examination as much as possible. If there is a large amount of adhesion in the abdomen or stomach, it is necessary to avoid contact or damage as much as possible to avoid inflammation and pain. After that, there is tumor resection, digestion, and evacuation.

**Figure 2:**
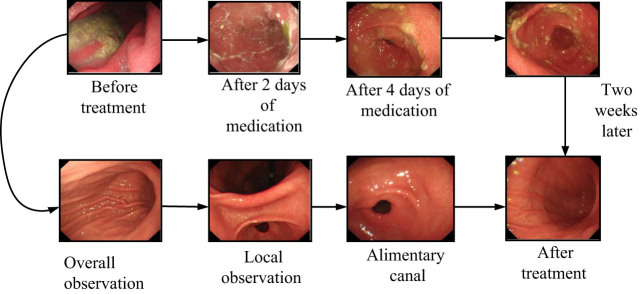
The treatment process of patients with NDS.

Targeted treatment methods should be adopted for the primary causes of neurogenic dyspepsia. Patients with acute peritonitis and abscesses should be treated with antibiotics, and attention should be paid to adjusting the electrolyte balance. When symptoms are severe, patients may experience stubborn decreased appetite, decreased bowel movements, recurrent swelling, abdominal distension, and other symptoms. After just completing the surgery, the patient should not have a lot of food in their stomach. There are also individual patients who overeat or drink too much water after surgery. If left unchecked, it can cause rapid swelling of the stomach again. The treatment process of patients with NDS is shown in Figure 2.

## 4 NDS experiment

### 4.1 Experimental description

One hundred patients with indigestion syndrome were selected and divided into an experimental group and a control group, with 50 cases in each group. Based on clinical diagnosis and pathological analysis, there were 22 males and 28 females in the control group. There were 26 males and 24 females in the experimental group. The data from the control and experimental groups were compared for statistical significance. The selection of experimental data is shown in Table 1.

**Table 1:** Selection of experimental data

Group	Number of male patients	Number of female patients	Therapeutic method
Control group	22	28	General medication treatment
Experimental group	26	24	Intelligent medical technology

Due to the tendency of patients to experience symptoms such as abdominal pain and bloating after eating, the patient was diagnosed with indigestion syndrome after professional examination. By analyzing the principles of organ diseases such as digestive system diseases and avoiding problems such as gastric cancer or gastrointestinal cancer, the control group was treated with ordinary drugs, while the experimental group was treated with intelligent medical technology. By observing the corresponding treatment, the control group and experimental group were analyzed and compared, and symptom scores were analyzed. Symptoms of indigestion include palpitations, vomiting, and abdominal distension. The score range was 1–9 points, with 1–3 points indicating no improvement or worsening of indigestion symptoms, 4–6 points indicating improvement of indigestion symptoms, and 7–9 points indicating disappearance of indigestion symptoms. Statistical Product and Service Solutions software was used to analyze and process the data, and standard deviation was used as the research indicator for analysis, with *P* < 0.05 indicating statistical significance.

### 4.2 Experimental results

#### 4.2.1 Treatment success rate

One hundred patients with NDS were selected. By setting up an experimental group and a control group, data analysis was conducted on the symptoms of palpitations, vomiting, and abdominal distension among the patients. Due to the use of ordinary drugs in the control group and intelligent medical technology in the experimental group, the treatment plan for long-term severe gastrointestinal diseases can be determined by analyzing the treatment status of patients with NDS in these two groups. The comparative analysis of treatment success rates between the control group and the experimental group is shown in Table 2.

**Table 2:** Comparative analysis of treatment success rates between the control group and the experimental group

Observation indicators	Cardiopalmus	Vomit	Abdominal distension	Adverse reactions score
Control group	34%	45%	52%	5
Experimental group	42%	52%	60%	8
Statistical significance	0.03			

Through the data analysis in Table 2, it was found that the success rate of treatment for palpitations in the control group was 34%, the success rate of treating vomiting symptoms was 45%, and the success rate of treating abdominal distension symptoms was 52%. The symptom score for adverse reactions to treatment was 5. After ordinary drug treatment, the symptoms of NDS in the control group of patients improved. The success rate of treating palpitations and vomiting symptoms in the experimental group was 42 and 52%, respectively; the success rate of treating abdominal distension symptoms was 60%, and the symptoms of NDS in the experimental group disappeared. By analyzing the data in Table 2, it is found that intelligent medical technology has a significant improvement effect on the treatment of symptoms of NDS in patients. Therefore, intelligent medical technology can play a role in eradicating symptoms of NDS and can be applied to clinical diagnosis and treatment.

#### 4.2.2 Time

By observing the food residue in the digestive tract of patients in the control group and experimental group after eating, as well as the disappearance time of comprehensive symptoms of neurodyspepsia, the data of the two groups of experiments were compared. Because the control group of patients with digestive dysfunction was treated with ordinary drugs, detailed observation is needed. The patients in the experimental group used intelligent medical technology to treat diseases faster, so their symptoms improved significantly. Two sets of data were analyzed by comparing the time of symptom disappearance and the residual food in the digestive tract. The comparison of various observation indicators is shown in Table 3.

**Table 3:** Comparison of various observation indicators between the experimental group and the control group

Observation indicators	Food residue	Disappearance time		
		Cardiopalmus	Vomit	Abdominal distension
Control group	2.0 ml ± 0.2 ml	3.2 h ± 0.4 h	2.8 h ± 0.2 h	2.7 h ± 0.1 h
Experimental group	1.5 ml ± 0.1 ml	2.6 h ± 0.2 h	1.8 h ± 0.1 h	1.6 h ± 0.2 h
Statistical significance	0.001	0.001	0.001	0.001

According to the data in Table 3, the residual amount of gastrointestinal food in the control group was 2.0 ± 0.2 ml, while the residual amount of gastrointestinal food in the experimental group was 1.5 ± 0.1 ml. After analysis, it was found that the experimental group had better treatment effects on patients through intelligent medical technology, and the disappearance time of comprehensive symptoms of neurodyspepsia was also shorter. The disappearance time of palpitation symptoms in the experimental group was 0.8 h shorter than that in the control group. After adopting intelligent medical technology, the vomiting symptoms of patients were also significantly improved, and the time for symptom disappearance was reduced by 1.1 h; the disappearance time of symptoms of abdominal distension reaction was also reduced by 1 h. After data processing, it was found that the overall data analysis value was *P* = 0.001 < *P* = 0.05, so the data analysis results of this experiment had statistical significance.

#### 4.2.3 Symptom points

**Figure 3:**
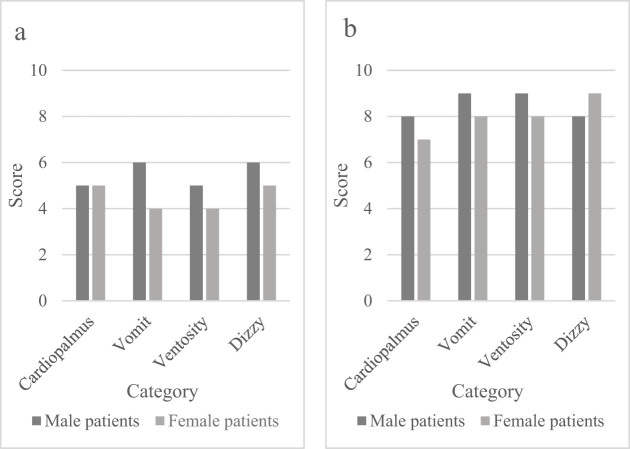
Symptom score values of the (a) control and (b) experimental groups.

According to the scoring principle of symptom integral values, the symptoms of male and female patients in both groups were scored, and the scorings of symptoms such as palpitations, vomiting, abdominal distension, and dizziness caused by indigestion were analyzed separately. The symptom score values are shown in Figure 3.

Figure 3a shows the symptom scores of the control group. According to Figure 3a, the overall symptom score of the control group was relatively low. Among them, the symptom score of male patients with palpitations was 5, the symptom score of vomiting was 6, the symptom score of abdominal distension was 5, and the symptom score for dizziness symptoms was 6; the symptom score for various symptoms in female patients was relatively low. Figure 3b shows the symptom scores of the experimental group. Through observation, it was found that the symptom score values of various indicators in male patients in the experimental group ranged from 8 to 9, while that of various indicators in female patients ranged from 7 to 9. Therefore, the integral value of symptoms of the experimental group treated with intelligent medical technology was higher, so intelligent medical technology can be applied to the clinical diagnosis and treatment of patients with comprehensive symptoms of neurodyspepsia.

#### 4.2.4 Factor scoring

**Figure 4:**
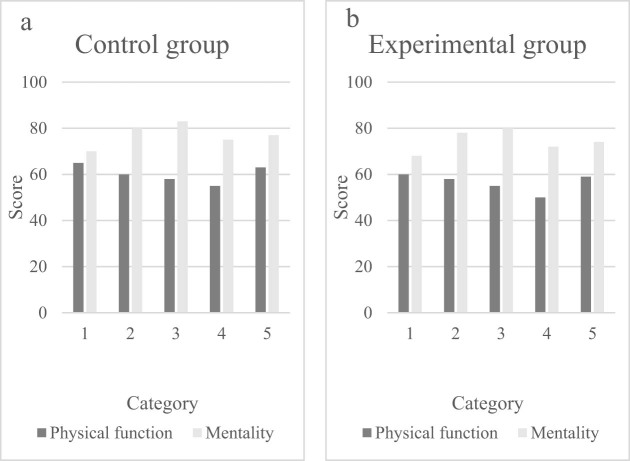
Scoring of influencing factors in the (a) control and (b) experimental groups.

By investigating the daily living conditions of 100 patients and dividing them into 5 groups, the physical function and mental state of patients with NDS were studied, and the quality of life of the patients was analyzed to determine the main influencing factors of the patient’s illness. A percentage system was used for scoring. The higher the score, the better the quality of life, indicating that this influencing factor has a smaller impact on NDS. The scoring of influencing factors is shown in Figure 4.

Figure 4a shows the score of influencing factors in the control group, while Figure 4b shows the score of influencing factors in the experimental group. The questionnaire scores of patients with comprehensive symptoms of neurodyspepsia in the control group ranged from 55 to 65 for physical function and from 70 to 83 for mental state. Through data analysis, it can be seen that the quality of life of the control group patients was higher, while the quality of life of the experimental group was lower. Therefore, the symptoms of the control group patients were milder, while the symptoms of the experimental group patients were more severe. It can also be seen that the score of physical function was lower than that of the mental state, indicating that physical function indicators had a greater impact on patients, while mental state indicators had a smaller impact. Through analysis, it was found that the physical function score of the experimental group was 6.3% lower than that of the control group. Therefore, in daily life, more attention should be paid to physical health. Good habits should be maintained, and the occurrence of indigestion syndrome should be reduced.

**Figure 5:**
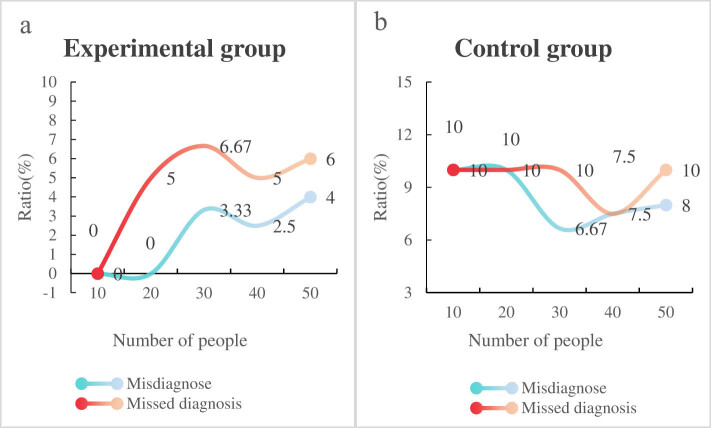
Misdiagnosis and missed diagnosis rates under two methods: (a) in the experimental group and (b) in the control group.

Finally, in order to verify the diagnostic accuracy of intelligent medical technology and other scholars’ research methods in the diagnosis of neurodigestive syndrome, this article compares it with the method in literature 2 and tests the misdiagnosis and missed diagnosis rates of the two methods for different patient numbers. The results are shown in Figure 5.

It can be seen from Figure 5(a) and (b) that the highest misdiagnosis rate for patients in the experimental group using intelligent medical technology is 4%; the highest missed diagnosis rate is 6.67%; the control group of patients in the experimental literature 2 method had the highest misdiagnosis rate and missed diagnosis rate of 10%. The misdiagnosis rate and missed diagnosis rate of patients in the experimental group were significantly lower than those in the control group, and the difference was statistically significant (*P* < 0.05). This indicates that the use of intelligent medical technology can improve diagnostic accuracy, which is of great significance for preventing neurodigestive syndrome.

## 5 Conclusions

By studying the clinical diagnosis and treatment plans of intelligent medicine for NDS, this article analyzed the application of intelligent medical technology in clinical practice and found that the application of intelligent medical technology can bring great convenience to the medical field. Through experimental data analysis, it was found that patients with NDS can be treated through intelligent medical technology with good results. In the experiment, the factors affecting patients suffering from neurological dyspepsia syndrome were analyzed to come up with a prevention program that people should be aware of in their daily lives. Both physical and psychological factors can have an impact on the symptoms of indigestion syndrome in patients, and diagnosis and treatment should be based on actual conditions. When there are problems with the defense mechanism of the digestive system, patients generally experience symptoms such as palpitations, vomiting, bloating, and dizziness and are prone to various diseases, which should be treated promptly. The application of intelligent medicine in the clinical diagnosis of NDS studied in this article can provide significant references for the application of intelligent medicine in other medical fields.
